# Novel nano-plasmonic sensing platform based on vertical conductive bridge

**DOI:** 10.1038/s41598-021-82899-6

**Published:** 2021-02-04

**Authors:** Hyo-Seung Park, Jongkil Park, Joon Young Kwak, Gyu-Weon Hwang, Doo-Seok Jeong, Kyeong-Seok Lee

**Affiliations:** 1 Center for Neuromorphic Engineering, Korea Institute of Science and Technology, Seoul, 02792 Korea; 2 Department of Materials Science and Engineering, Hanyang University, Seoul, 04763 Korea

**Keywords:** Nanophotonics and plasmonics, Sensors and biosensors

## Abstract

A novel nano-plasmonic sensing platform based on vertical conductive bridge was suggested as an alternative geometry for taking full advantages of unique properties of conductive junction while substantially alleviating burdens in lithographic process. The effects of various geometrical parameters on the plasmonic properties were systematically investigated. Theoretical simulation on this structure demonstrates that the presence of vertical conductive bridge with smaller diameter sandwiched between two adjacent thin nanodiscs excites a bridged mode very similar to the charge transfer plasmon and exhibits a remarkable enhancement in the extinction efficiency and the sensitivity when the electric field of incident light is parallel to the conductive bridge. Furthermore, for the electric field perpendicular to the bridge, another interesting feature is observed that two magnetic resonance modes are excited symmetrically through open-gaps on both sides of the bridge together with strongly enhanced electric field intensity, which provides a very favorable environment as a surface enhanced Raman scattering substrate for fluid analysis. These results verify a great potential and versatility of our approach for use as a nanoplasmonic sensing platform. In addition, we demonstrated the feasibility of fabrication process of vertical conductive bridge and high tunability in controlling the bridge width.

## Introduction

A localized surface plasmon (LSP) excited in metallic nanostructures, sometimes called nanoplasmon, has received tremendous attention due to the peculiar optical properties; resonant optical scattering and local field enhancement. These properties along with the highly sensitive response of its resonance condition to a change in local environment have triggered extensive studies on biochemical sensors^1–4^. Nanoplasmonic sensors are especially useful for the detection of target molecules with a very small molecular weight and trace amounts of components when compared with the conventional surface plasmon resonance (SPR) sensors because the distribution of local electric field (E-field) is confined within several to tens of nanometers from the metal surfaces, while the E-field decay length of SPR sensor reaches hundreds of nanometers or longer^1,5^.

Since the plasmonic properties can be profoundly modified by structural design, various shapes and geometries have been explored to get a better control over those properties^6–8^. In terms of tunability and sensitivity, anisotropic structures have shown better performance over isotropic ones. Especially, one-dimensional prolate spheroids such as Au and Ag nanorods have long been a preferred shape to the spherical and oblate ones^9,10^. A longitudinal mode excited along the long axis accounts for their superior properties^9,10^.

More interesting features are found in dimer structure^11–13^. When the plasmonic particles are placed very close to each other, their local fields become overlapped and a dipolar coupling begins to start. This leads to a red-shift of resonance wavelength exponentially dependent on the decreasing interparticle separation and a generation of hot spot in the nanogap. The most dramatic changes occur when the two particles get into contact. As reported by Jensen, et al.^14^, dipolar interaction between the two particles change its character when they are conductively connected by a small junction. A dipolar resonance mode abruptly splits into two discrete modes. One appears jumped up to a long wavelength region and the other occurs at a shorter wavelength. Comparison with theoretical simulations revealed that the former is ascribed to a dipole mode related with the conductive junction formation and the latter was initially referred to as a quadruple mode^12,15^ but later more accurately recognized as a screened bonding dimer plasmon (SBDP) mode^16,17^. The largely red-shifted dipolar mode is quite sensitive to the extent of overlap and changes its resonance wavelength inversely proportional to it. As the particle pair merges further, such an abnormality disappears and overall behavior begins to resemble that of single prolate particle.

Other reports, based on a well fabricated nanodisc pairs with controlled separation and overlap, also experimentally verified the large split of plasmon mode upon contact and a gradual blue-shift of the dipolar mode as they are merged together^15,18^. According to Berkovitch et al.^19^, nanoparticles with concave cross-section exhibit wide tunability similar to that observed in conductive junction. They extended their work by applying a narrow shortening wire between plasmonic dimers separated from each other, i.e. forming a conductive bridge between them^20^. Enhancement in local field and tunability was observed to be more significant compared with the effect of concavity.

Such dimer geometries with a conductive junction have been dominantly fabricated using e-beam lithography (EBL). Although EBL is advantageous in generating geometrically complex nanoscale structures, the precise control of shallow planar junction is still hard to achieve and being a part of great challenges. In this study, we suggest an alternative geometry for taking full advantages of unique properties of conductive junction while substantially alleviating burdens in lithographic process. Our approach is based on a three-layered structure of metallic nanodiscs with oblate shape. The key idea is to place a metal nanodisc in the middle whose diameter can be tuned to form a neck which acts as a vertical conductive bridge (VCB). The effect of geometrical parameters defining the VCB such as a neck width and length on its plasmonic properties was theoretically investigated and reported here in terms of local field enhancement, tunability, and sensitivity. An effective way of exciting and utilizing the VCB mode is proposed as well. In addition, the characteristics of the magnetic resonance modes that occur when electric field of incident light is perpendicular to the conductive bridge were analyzed in comparison with that of conventional metal–insulator-metal (MIM) nano-resonator structure.

## Results and discussion

### Charge transfer plasmon mode in overlapped dimer

**Figure 1:**
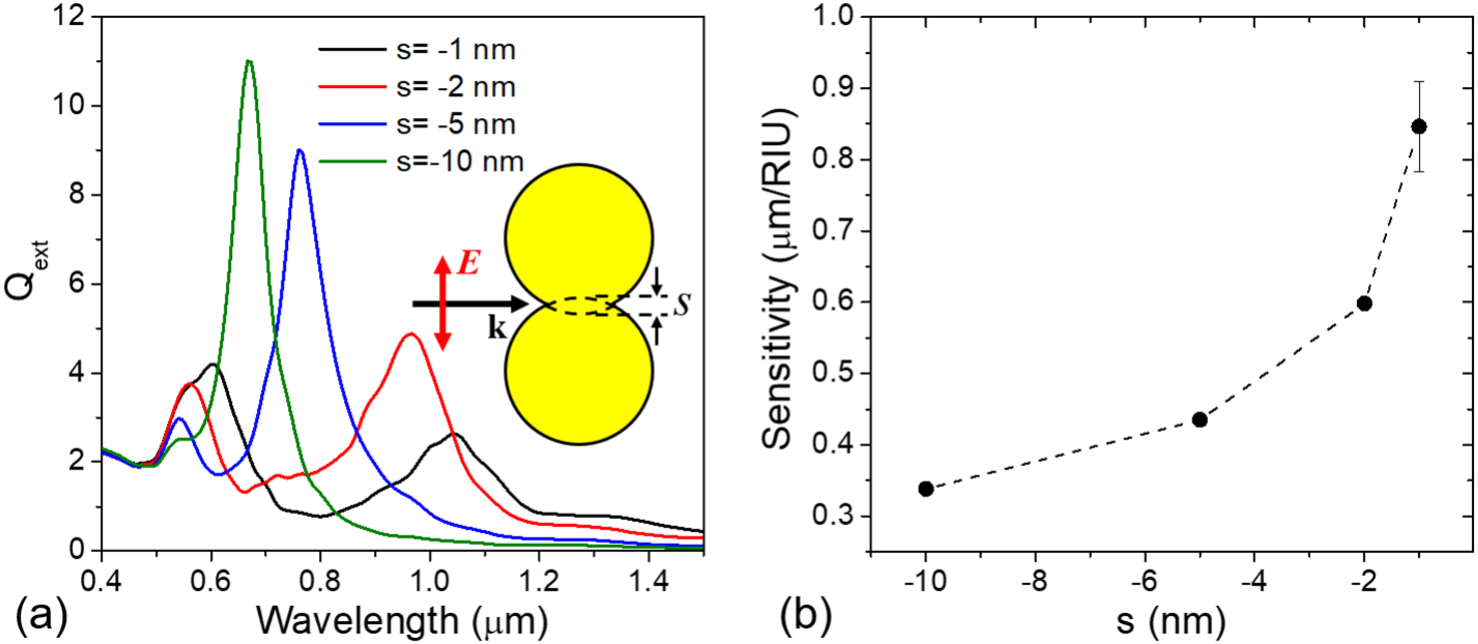
(**a**) Calculated extinction efficiency as a function of separation between the two identical Au spheres. Negative sign in the separation indicated the spheres are overlapped and merged. Inserted is the schematic configuration. (**b**) Dependence of the conductively coupled dimer’s sensitivity on the extent of overlapping.

Before going into the details of VCB geometry, we examined a basic model system consisting of Au nanosphere dimer, as shown in Fig. 1, in order to have a grasp of the spectral evolution of conductive junction mode and its plasmonic properties. Theoretical simulation was carried out using a discrete dipole approximation (DDA) method^14^. We employed the DDA code developed by Draine and Flatau^21^, and characterized the case with fixed target orientation where the incident light is polarized parallel to the dimer axis. Details of DDA calculations have been described elsewhere^22^.

Figure 1a shows how the extinction efficiency spectra evolve as the dimer is merged with each other. Here, two Au nanospheres of 40 nm radius were assumed to be in water environment. The negative value of separation parameter, *s*, means the overlap between the two particles to that extent. Once the dimer is connected through a conductive junction, so-called charge transfer plasmon (CTP) mode^23,24^ emerged far apart in the long wavelength region. As the overlap increased further, i.e.* s* changed its value from − 1 nm to − 10 nm, the dipolar CTP mode rapidly shifted toward shorter wavelength region and finally merged with the SBDP mode. From the Fig. 1a, it is obvious that the CTP mode provides a wide range control of its spectral position depending on the extent of overlap. Since the response is very sensitive, however, a small fluctuation may yield a large amount of spectral shift which makes precise tuning hard to control.

We also investigated the sensitivity of CTP mode as a function of overlap as shown in Fig. 1b. Here, the sensitivity is defined as a resonance wavelength shift in response to the refractive index change of surrounding medium. The sensitivity exhibits an inverse proportional relationship with the extent of overlap and is likely to reach maximum at the moment of contact. Note that the sensitivity of CTP mode is remarkably enhanced when compared with that of isolated Au nanosphere limited below 200 nm/RIU^8,9,25^. The error bar means the standard error of linear regression performed to determine the sensitivity from the resonance wavelength versus environmental refractive index plot and it is negligible except for the case of s = -1 nm.

### Novel approach via a formation of vertical conductive bridge and its characteristics

**Figure 2:**
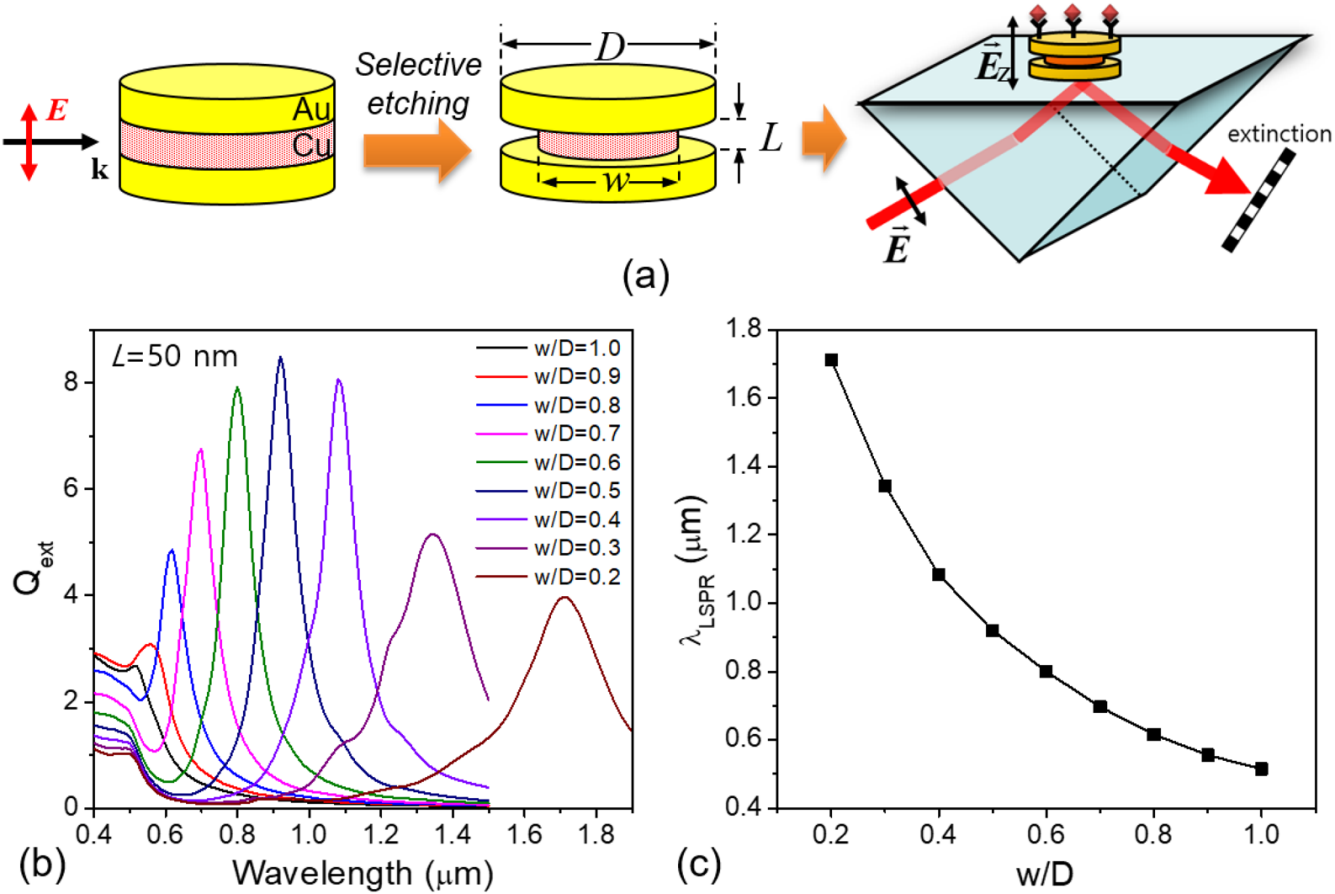
(**a**) Schematic of nanoplasmonic sensing platform with a vertical conductive bridge and its fabrication via selective etching process. (**b**) Spectral tunability of Au nanodiscs bridged with vertical conductive Cu junction and (**c**) the dependence of its resonance wavelength on the fractional bridge width.

Figure 2a shows a schematic of the proposed nanostructure with a VCB and its fabrication process. Initially, a three-layered metallic nanodisc is fabricated by inserting an intermediate Cu layer which has a different corrosion reactivity from Au constituting the top and bottom layers. Then, a selective etching proceeds in acidic environment so as to form a trenched waist which acts as a VCB. The bridge width, *w*, can be adjusted in a controllable manner by carefully designing the etching times and conditions. The bridge length, *L*, is determined by the thickness of Cu layer.

In this platform, the excitation of dipolar CTP mode due to the conductive bridge can be achieved by applying an incident light such that the direction of an electric field is parallel to the conductive bridge and perpendicular to the disc surface. Evanescent field coupling provides a very facile way of doing this. Referring to a schematic shown in right side of Fig. 2a, a p-polarized incident light undergoes a total internal reflection at the hypotenuse of prism creating an evanescent field at the interface whose dominant polarization is perpendicular to the interface.

The optical absorption and scattering properties were calculated by a DDA simulation for this case with varying the bridge width and length. Figure 2b shows the spectral evolution of total extinction efficiency calculated before and after the formation of VCB. The top and bottom Au nanodiscs have the same dimensions. Their diameter and length are set to 200 nm and 20 nm, respectively. Whereas, the Cu bridge is assumed to have a varying width from 200 to 40 nm. The bridge length was fixed to be 50 nm. Before the trenched waist is formed, i.e. when the diameter of intermediate Cu disc is 200 nm, the extinction efficiency exhibits a small single transverse mode of an oblate geometry. However, as the width of Cu bridge decreases forming the trenched waist, the resonance mode rapidly red-shifts and the magnitude of extinction efficiency undergoes a quite large enhancement. Besides, the spectral profile shows a very sharp and narrow linewidth except where the bridge width is too narrow.

Figure 2c shows how much the resonance wavelength actually shifts depending on the fractional width of bridge with regard to the diameter of Au nanodiscs. It is clear that by reducing only the width of bridge, it is possible to tune the resonance wavelength over a very broad range. These characteristics are very similar to that of CTP mode observed in the overlapped dimer shown in Fig. 1 and even seem to provide better tunability, not having the extreme vulnerability to a small fluctuation in the region of contact point. These can be further modified by a control of geometrical parameters and a proper selection of bridge metal such as Ag.

**Figure 3:**
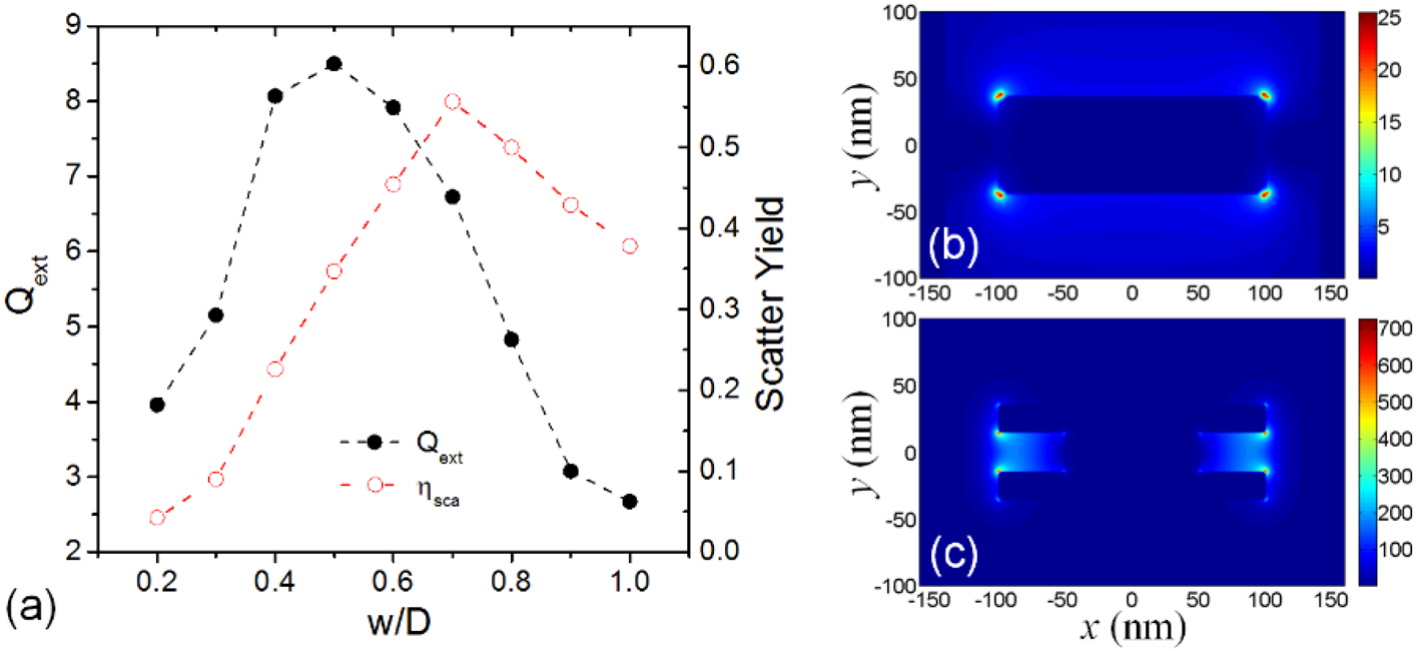
(**a**) Dependence of magnitude of extinction efficiency and scattering quantum yield on the fractional width of bridge, and calculated distribution of E-field intensity around the multilayered disc (**b**) before and (**c**) after the Cu bridge formation at each resonance maximum.

Figure 3 summarizes the dependence of magnitude of extinction efficiency and a radiative scattering quantum yield on the fractional bridge width. Here, the scattering quantum yield *η* are defined as

1 $$\eta ={\left.\frac{{Q}_{sca}}{{Q}_{ext}}\right|}_{Res}$$

which is the ratio of scattered efficiency to the total extinction at each resonance maximum^22^. Both the extinction magnitude and the scatter yield show a peak curve, although there is a little difference in its position. A trenched waist formation gives rise to a substantial increase especially in the total extinction which reaches a maximum around *w/D* of 0.5 and begins to fall with more severe necking. Early descent in scattering yield might be ascribed to an immoderately distorted distribution of local electric field (E-field) which deteriorates coherent oscillation of surface electrons and makes the nanodisc absorptive in nature. Nonetheless, it should be noted that the scattering quantum yield is considerably enhanced when creating a shallow trench, i.e. it increases from 0.38 for the case of *w/D* = 1.0 to 0.56 when *w/D* = 0.7 while the volume of nanodisc (total number of metal atoms) decreases.

E-field intensity distributions at resonance wavelengths were computed in the axial cross-section of VCB nanodiscs by a finite-difference-time-domain (FDTD) method using a commercial software (Lumerical, FDTD Solutions^26^) and plotted as a contour map in Fig. 3b,c for the cases of *w/D* = 1.0 and 0.5, respectively. Without a VCB, near-field intensity distribution follows a simple dipolar behavior of general transverse mode of circular disc, while the trenched disc shows a substantial redistribution of surface charge passing though the VCB which reduces the restoring force of oscillating electrons and contributes to the large red-shift and the E-field enhancement. Note that the VCB nanodisc of *w/D* = 0.5 exhibits about 28 times higher near-field intensity than the disc without bridge. Moreover, the hot-spots of electric field concentrated in the nanogap near the conductive bridge can be beneficially applied to a field enhanced spectroscopy.

**Figure 4:**
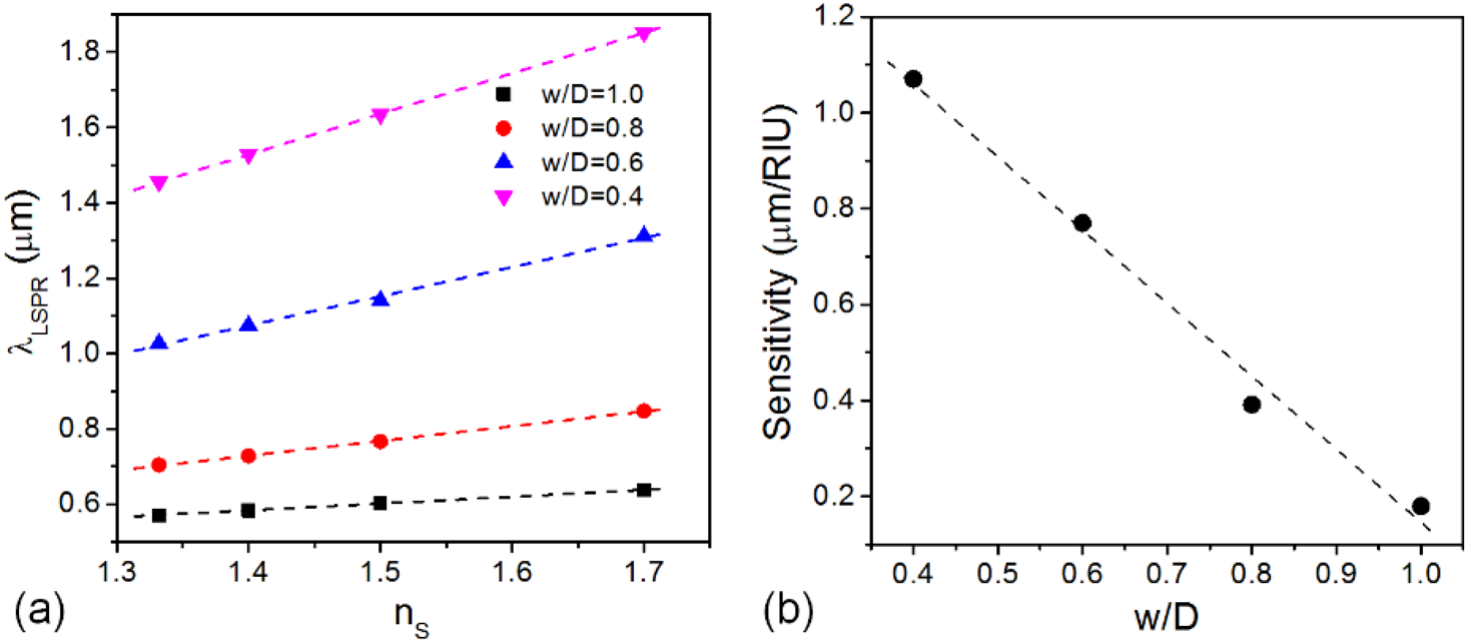
(**a**) Resonance wavelength of VCB nanodiscs with variable bridge widths as a function of the refractive index of surrounding medium and (**b**) the dependence of sensitivity of VCB nanodiscs on fractional bridge width.

The spectral response of VCB nanodiscs to an environmental change and its dependence on the fractional bridge width were also investigated using the DDA simulation. Figure 4a summarized the calculated resonance wavelengths as a function of the refractive index of surrounding medium (*n*_s_), assuming it is gradually varied from 1.332 to 1.7. Without regard to the bridge width, the resonance wavelength is linearly red-shifted with increasing *n*_s_, while the proportional constant, i.e. the slope of line representing the sensitivity is noticeably getting higher as the bridge width decreases.

Figure 4b summarizes the sensitivity of VCB nanodiscs as a function of fractional bridge width. Note that there is a strong linear negative correlation between the sensitivity and the fractional bridge width. In other words, the narrower the bridge width is, the higher sensitivity can be obtained. The sensitivity enhancement is remarkable and the value of sensitivity amounts to 1.07 μm/RIU when *w/D* = 0.4, about 5–6 times larger than that of normal Au nanosphere, which implies the VCB nanodisc is very promising as a nanoplamonic sensing platform.

**Figure 5:**
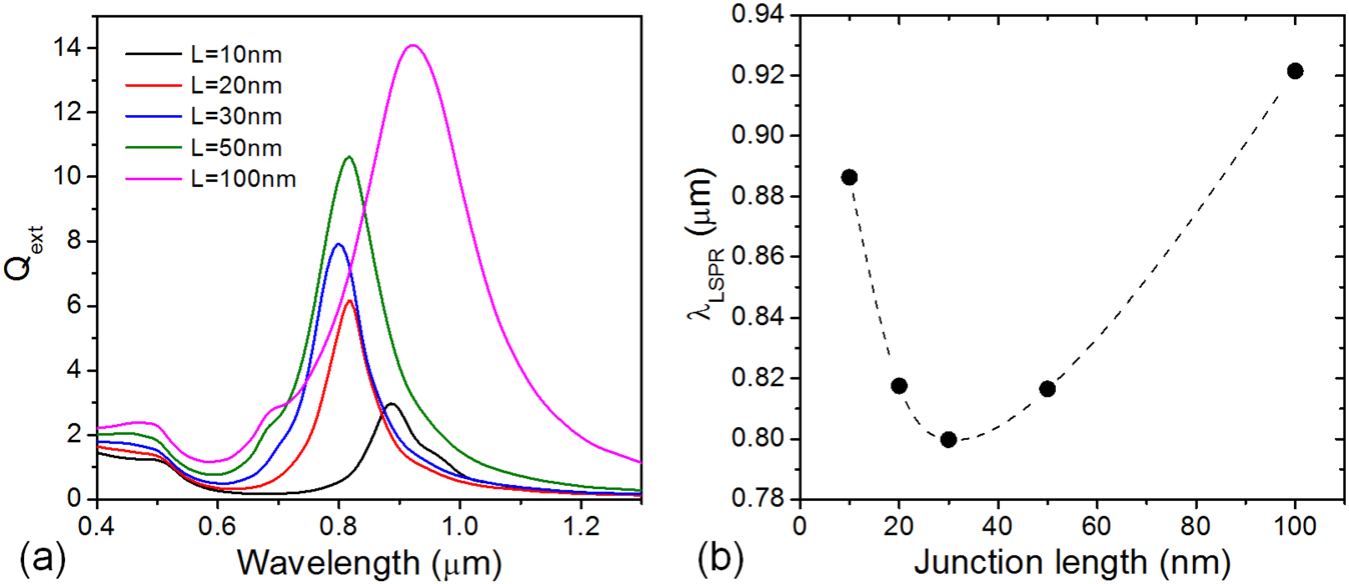
Effect of VCB length change on the (**a**) total extinction efficiency spectra and (**b**) their resonance wavelength.

The effect of bridge length *L* on the properties of CTP mode was also investigated with the VCB nanodisc of *w/D* = 0.6. Figure 5 shows how the extinction efficiency spectra depend on the bridge length. The length of the Cu conductive bridge was varied from 10 to 100 nm. It appears from the Fig. 5 that the magnitude of extinction efficiency monotonically increases with the bridge length, while the resonance wavelength varies in somewhat complex manner. The resonance wavelength was summarized in Fig. 5b as a function of bridge length and exhibits a minimum at *L* = 30 nm. In either case of being shorter or longer than 30 nm, it is observed that the resonance wavelength begins to increase. The red-shift observed with decreasing bridge length below 30 nm might be dominated by a strong near-field interaction occurred between the top and bottom Au nanodiscs through the nanogap formed in trenched edge sides. The close proximity leads to more red-shifts. However, excessively distorted distribution of induced polarization is thought to limit the magnitude of total extinction.

On the other hand, as the conductive bridge length extends well beyond 30 nm, the overall trend in resonance properties looks gradually getting similar to that of longitudinal mode of prolate particles. The increase in bridge length causes the red-shift in resonance wavelength as well as the strong enhancement in total extinction. Moreover, the scattering quantum yield is significantly improved as the bridge length increases further (not shown here).

**Figure 6:**
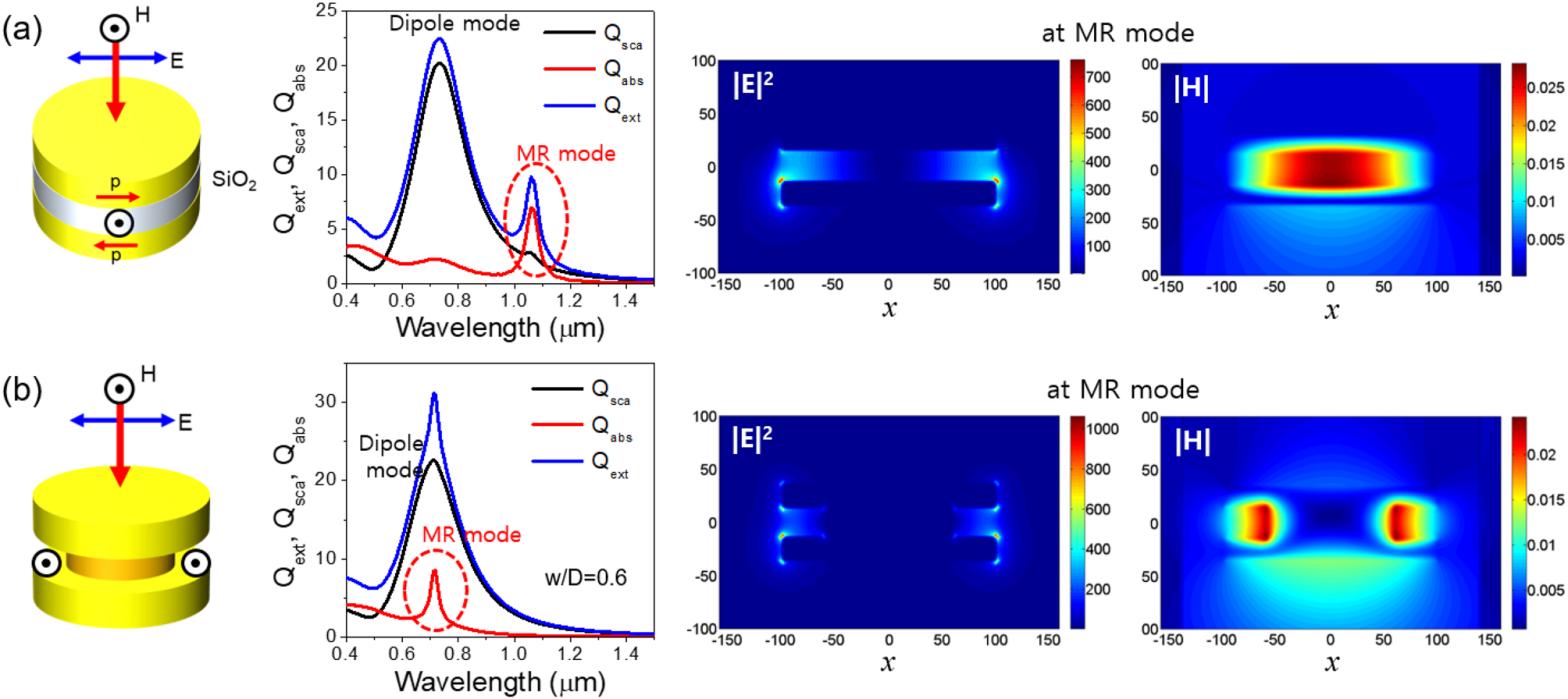
(**a**) Schematic configuration of conventional metal–insulator-metal (MIM) nanodisc and its extinction efficiency spectra from DDA simulation, cross-sectional view of E-field intensity and magnetic field distribution in the x–z plane across the disc center calculated at the spectral position of magnetic mode using FDTD method. (**b**) Schematic configuration of VCB nanodisc and its extinction efficiency spectra from DDA simulation, cross-sectional view of E-field intensity and magnetic field distribution in the x–z plane across the disc center calculated at the spectral position of magnetic mode using FDTD method. The incident light is assumed to be polarized in the x-direction.

Figure 6 compares the characteristics of magnetic resonance modes^27–29^ excited when the electric field of incident light is parallel to the surface of metal nanodiscs for the conventional MIM configuration (Fig. 6a) and the VCB nanodiscs (Fig. 6b). Here, the diameter and thickness of Au nanodiscs and the spacer length were 200 nm, 20 nm, and 50 nm, respectively. For the VCB nanodisc, *w/D* was 0.6. In both cases, total extinction spectra consisting of scattering and absorption efficiencies were calculated first to identify the magnetic resonance (MR) mode. The MR mode is generally absorption dominant due to the impedance matching effect, and exhibits a narrower and sharper resonance curves in MIM nanodiscs. Therefore, it is thought that the sharp absorption efficiency peaks shown in Fig. 6 are ascribed to the MR mode. On the other hand, the electric dipole modes exhibit scattering-dominant characteristics mainly due to the volumetric radiative capacity related with the nanodiscs size. While the MR mode in MIM nanodiscs is clearly distinguished in the low energy side, it appears to be superimposed with the electric dipole mode curve in the case of VCB nanodiscs. Although not shown here, it should be noted that the MR mode in the VCB can be tuned to a lower energy side by reducing the bridge width. In conventional MIM structure, strong mode confinement due to the magnetic dipole resonance is observed inside a dielectric spacer. Interestingly, on the other hand, two magnetic resonance modes are observed to be excited symmetrically with respect to the *x* = 0 plane through the open-gaps on both sides of the bridge in the VCB nanodiscs together with stronger E-field enhancement, which provides a very favorable environment as a surface enhanced Raman scattering substrate for fluid analysis. It should be noted that analytes in fluids to be detected are freely accessible to the position where the strong field enhancement occurs, while it is blocked considerably in the conventional MIM structure.

### Preliminary demonstration of VCB nanodisc fabrication process

**Figure 7:**
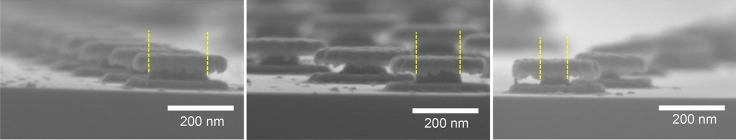
SEM images of VCB nanodiscs array which proves the feasibility of the fabrication process suggested.

In Fig. 7, we show a series of cross-sectional scanning electron microscope (SEM) images which present a preliminary result on the fabrication of VCB nanodiscs and demonstrate the feasibility of our suggested process. The arrays of multilayered nanodiscs consisting of Au(20 nm)/Cu(50 nm)/Au (20 nm) layers were fabricated on a Si substrate just for the SEM analysis using a modified photonic nanojet lithography^30^. The creation of trenched bridge was made by immersing the sample in an alkaline developer for selective etching and the width of bridge was controlled by the immersion time. Figure 7 verifies the feasibility of fabrication process and the high tunability in controlling bridge width. From the left image in Fig. 7 to the right, the immersion time was 80 min, 90 min, and 100 min, respectively. It is also inferred from the SEM images of Fig. 7 that the e-beam evaporated atoms are not perfectly collimated and reach to the patterned PR with an angular divergence, which causes the multilayered nanodisc to have oblique sidewalls. As a results, it is thought that the upper Au layer slightly covers the sidewall of the Cu layer in the middle and forms a “torn skirt” in the process of etching out the Cu layer. This could be easily improved by employing a highly collimated e-beam evaporation system.

## Conclusions

In summary, we presented a novel platform of highly sensitive and tunable LSPR sensor based on a vertical conductive bridge as a better alternative to the planar geometry of conductively touching dimers in terms of plasmonic properties and facileness of fabrication process. Theoretical simulation on this structure confirmed that the presence of Cu VCB with a smaller width sandwiched between two adjacent Au nanodiscs allows the excitation of a CTP mode and exhibits a remarkable enhancement in tunability, local field, and sensitivity. It was found that the bridge width has a dominant effect on those plasmonic properties in such a way that narrowing the bridge width leads to a monotonic increase of resonance wavelength and sensitivity, while the local field enhancement and the scattering quantum yield exhibited a maximum in a moderate bridge width. In our model structure, the moderate bridge width of 50% yielded a peak intensity of E-field about 28 times higher than the case without bridge. The sensitivity, for example, at *w/D* = 40% amounted to 1.07 μm/RIU, 5–6 times larger than that of normal Au nanosphere. This implies the VCB nanodisc is very promising as a nanoplasmonic sensing platform. We also suggested a convenient sensor configuration which employs an evanescent field coupling method optimized for the excitation of CTP mode from the VCB nanodiscs and proved the feasibility of forming the VCB via a selective etching process. Furthermore, for the incident light polarized perpendicular to the bridge, it is confirmed that two magnetic resonance modes are excited symmetrically with respect to the *x* = 0 plane through the open-gaps on both sides of the bridge together with strongly enhanced E-field intensity, which provides a very favorable environment as a surface enhanced Raman scattering substrate for fluid analysis.

## Methods

### Theoretical simulation

Optical extinction spectra of the Au nanosphere dimers and VCB nanodiscs were calculated using a DDA method. We employed the DDA code developed by Draine and Flatau^21^, and characterized the case with fixed target orientation where the incident light is polarized parallel to the dimer axis. The number of dipoles *N* approximating the target geometry was optimized to be a number for which the numerical results converge to a certain value and change little by further increase in *N* while sufficiently well reproducing the target surfaces. Near field distribution of electromagnetic field across the VCB nanodiscs was calculated using a FDTD method (Lumerical, FDTD Solutions). A total-field/scattered-field (TFSF) plane-wave source was used and an override mesh region with a 2 nm mesh size in all directions was added throughout the TFSF region. The perfectly matched layer boundary conditions (PML) were applied to enclose the simulation region. The simulation time was set to 150 fs. The optical constants for Au and Cu were taken from Johnson and Christy^31^.

### Fabrication of VCB nanodiscs

The VCB nanodiscs were fabricated using a two-step process consisting of a photonic nanojet lithography for the formation of Au/Cu/Au multilayered nanodiscs and a subsequent Cu etching process for the creation of trenched bridge. A dual-layer resist stack was adopted to facilitate the lift-off process. A thin positive photoresist (AZ5214E diluted 1:4 with AZ1500 thinner) was spin-coated at 7000 rpm for 60 s on the undercut-forming layer (Shipley, LOL2000) pre-coated on a Si substrate. Then, the resist was baked on a hotplate at 90 °C for 90 s. The thickness of the LOL and the PR layers was almost the same at 150 nm. A hexagonal close-packed monolayer of polystyrene beads (Polyscience Inc.) of 1 μm diameter was self-assembled on the resist layer using a drop coating method and exposed to UV h-line (405 nm) for 12 s using a mask-aligner (Suss MicroTec, MA6). To avoid undesirable patterning in a non-focused region, the light power density was adjusted to be as low as 1.2 mW/cm^2^ by inserting a neutral density filter with an optical density of 1. After UV exposure, the PS beads were removed by ultrasonication in DI water and the samples were developed in AZ300 MIF developer to obtain a desirable undercut in the LOL layer below the PR hole. Then, the multilayers of Ti(5 nm)/Au(20 nm)/Cu(50 nm)/Au(20 nm) were e-beam evaporated sequentially on the patterned resist. Finally, both the lift-off process and selective Cu etching were carried out simultaneously by immersing the sample in an alkaline developer (Microposit, CD-30) of pH 12, which produced the array of VCB nanodiscs. The width of bridge was controlled by the immersion time.
